# *Mycoplasma gallisepticum* escapes the host immune response via gga-miR-365-3p/SOCS5/STATs axis

**DOI:** 10.1186/s13567-022-01117-x

**Published:** 2022-12-05

**Authors:** Yingjie Wang, Yun Han, Lulu Wang, Mengyun Zou, Yingfei Sun, Huanling Sun, Qiao Guo, Xiuli Peng

**Affiliations:** Key Laboratory of Agricultural Animal Genetics, Breeding and Reproduction, Ministry of Education, Huazhong Agricultural University, Hubei 430070 Wuhan, China

**Keywords:** *Mycoplasma gallisepticum*, Gga-miR-365-3p, SOCS5, immune escape, JAK/STAT

## Abstract

A disruption in the expression of gga-miR-365-3p was confirmed in the *Mycoplasma gallisepticum* (MG)-infected Chicken primary alveolar type II epithelial (CP-II) cells based on previous sequencing results, but the role it plays in the infection was unclear. In the present study, we demonstrate that MG evaded cellular host immunity via a gga-miR-365-3p/SOCS5-JAK/STATs negative feedback loop. Specifically, we found that at the initial stage of MG infection in cells, gga-miR-365-3p was rapidly increased and activated the JAK/STAT signaling pathway by inhibiting SOCS5, which induced the secretion of inflammatory factors and triggered immune response against MG infection. Over time, though, the infection progressed, MG gradually destroyed the immune defences of CP-II cells. In late stages of infection, MG escaped host immunity by reducing intracellular gga-miR-365-3p and inhibiting the JAK/STAT pathway to suppress the secretion of inflammatory factors and promote MG adhesion or invasion. These results revealed the game between MG and host cell interactions, providing a new perspective to gain insight into the pathogenic mechanisms of MG or other pathogens. Meanwhile, they also contributed to novel thoughts on the prevention and control of MG and other pathogenic infections, shedding light on the immune modulating response triggered by pathogen invasion and their molecular targeting.

**Graphic Abstract:**

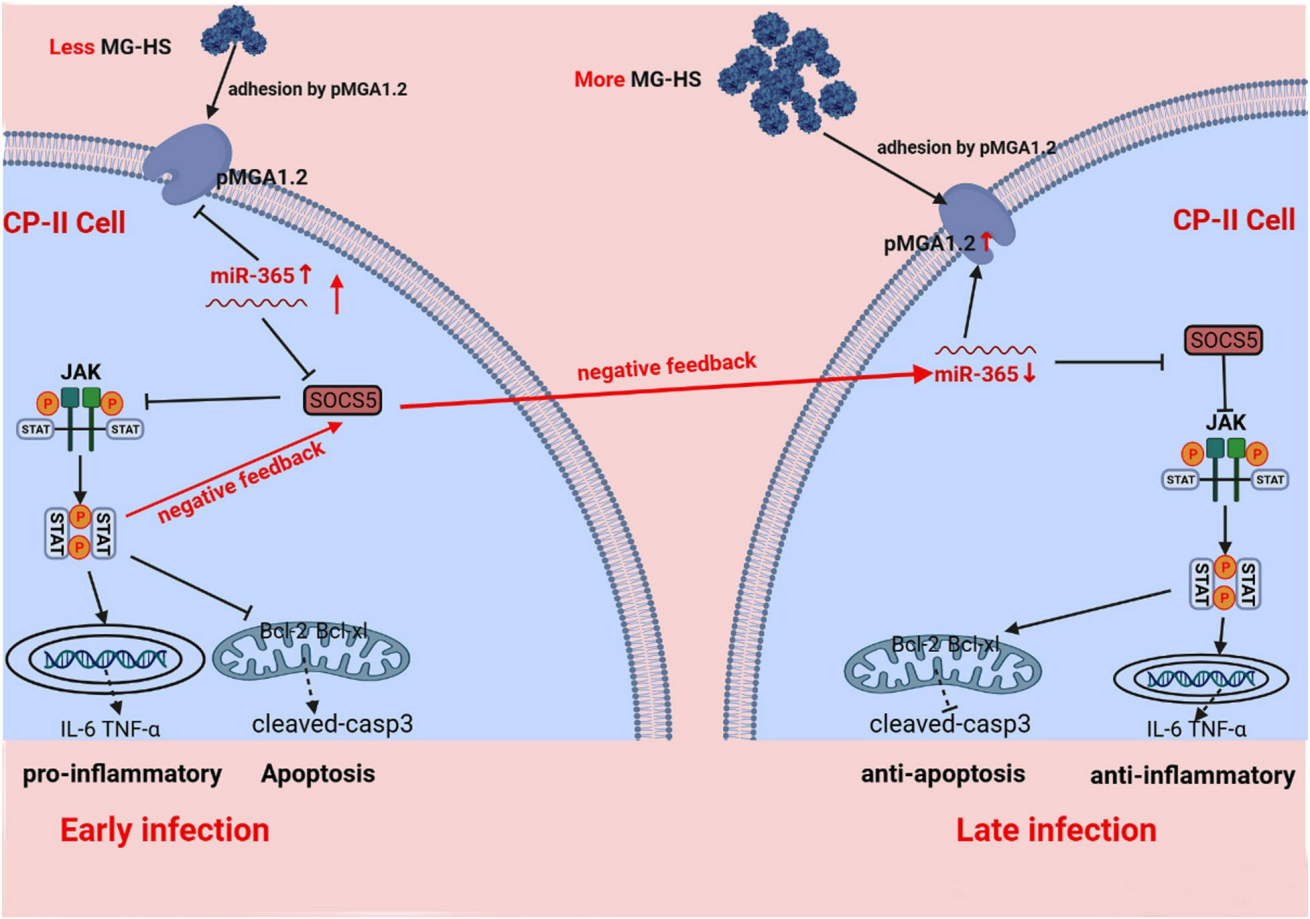

## Introduction

*Mycoplasma gallisepticum* (MG), an avian pathogen, is known to induce chronic respiratory disease in chickens, causing considerable economic losses to the poultry industry [1–3]. Current studies have demonstrated that MG adheres to the host respiratory mucosa through its membrane proteins (mainly GapA, PvpA, CrmA, pMGA1.2 and MGC2/3), while attenuated strains lacking adhesion proteins are unable to bind to host cell receptors to establish infection [4–8]. There exist multiple methods for MG to evade the host immunity. For example, several dozen pMGA gene families of MG adhesion proteins can escape the host immunity by switching the expression of different pMGA genes to produce antigenic variants [9, 10]. The fine mechanism of MG evasion immunity remains to be further elucidated.

In recent years, studies have shown that miRNA have an important role to play in the regulation of various avian diseases [11, 12]. gga-miR-1249 and gga-miR-485 target PB proteins to inhibit avian influenza virus replication [13]. In spleen tumors of chickens infected with Marek disease virus (MDV), the expression of gga-miR-15b, which targets the tumor suppressor ATF2, was significantly decreased. Suppressing gga-miR-219b expression can inhibit the expression of MDV oncogene Meq [14]. Furthermore, in the study of infectious bursal disease, miRNA were found to regulate avian innate immunity through SOCS proteins. For example, gga-miR-155 can inhibit IBDV replication by targeting SOCS1 and TANK [15]; gga-miR-130b inhibits IBDV replication by targeting a specific sequence of IBDV segment A and enhancing IFN-β expression by binding to host SOCS5 [16]; gga-miR-454 inhibits IBDV replication by binding to a specific sequence of IBDV segment B and targeting SOCS6 to enhance IFN-β expression [17]. Our previous findings showed that 45 and 68 miRNA were differentially expressed in lung tissue on the third and tenth day after MG infection of chick embryos, respectively, and that these miRNA targeted 6290 and 7181 genes, respectively [18]. Some differentially expressed miRNA play regulatory roles in MG infection through Toll-like receptors, NF-*κ*B and MAPK signaling pathways [19, 20], while the function of gga-miR-365-3p remains to be explored.

The JAK/STAT pathway is an ubiquitously expressed intracellular signaling pathway [21], and many cytokines and growth factors of this signaling pathway [22], regulate immune adaptability, inflammation, and apoptosis [23]. The suppressor of cytokine signaling (SOCS), also known as STAT-mediated STAT inhibitor protein (SSI), is produced by activation of the JAK/STAT pathway [24]. SOCS proteins inhibit the JAK/STAT pathway signal in turn through various ways, including combining with the phosphorylated JAK protein, binding to the phosphorylated region of jak receptor or inhibiting the activity of the jak receptor [25]. The presence of SOCS creates a negative feedback loop in JAK/STAT signaling, avoiding an extreme immune response [26].

Because of the immunomodulatory role of SOCS, it is commonly accepted that infectious microorganisms can manipulate host SOCS proteins to evade immunity [27]. Numerous data suggest that a variety of pathogenic bacteria, including *E. coli*, *Chlamydia pneumoniae* and S*almonella enterica*, function to induce transcription and/or protein expression of SOCS1 or SOCS3 using multiple signaling pathways (including STAT1, STAT3, MAPK and NF-κB) to control the inflammatory response [28]. In addition, it has also been shown that the antiviral function of IFN can be inhibited by SOCS3 [29].

The purpose of this study was to investigate the role of gga-miR-365-3p/SOCS5 in MG infection and their molecular mechanisms.

## Materials and methods

### CP-II cell culture and treatment

Chicken primary alveolar type II epithelial (CP-II) cells were collected according to our established method [30, 31]. All cells were cultured in Dulbecco modified Eagle medium (DMEM) supplemented with 15% fetal bovine serum (FBS) (Gibco, Shanghai, China) in a carbon dioxide cell incubator with 5% CO_2_ at 37 ℃. Lipofectamine 2000 (Invitrogen Life Technologies, USA) was used to transfect oligonucleotides and/or plasmids into cells. It is worth pointing out that MG-HS was used to challenge the cells at 24 h after transfection.

### Mycoplasma strains

MG-HS, a virulent strain, was isolated previously from a chicken farm in Hubei Province of China [32]. The concentration of viable *Mycoplasmas* in a suspension was then determined by a color-changing unit (CCU) assay that was reported in detail in our previous studies [33]. The concentration of MG-HS in this study was 10^9^ CCU/mL.

### DNA primers and RNA oligonucleotides

In this experiment, total sequences of DNA primers that were adopted are presented in Table 1. In addition, All RNA oligonucleotides were designed and synthesized by GenePharm (Shanghai, China). The RNA oligonucleotide sequences are shown in Table 2.

**Table 1:** **Sequences of DNA primers**

Name	Primer Sequence (5′-3′)
Primers for CDS Cloning	
SOCS5-CDS-F	TAGCGTTTAAACTTAAGCTTATGGATAAAGTGGGAAAGATGT
SOCS5-CDS-R	CACAGTGGCGGCCGCTCGAGTTACTTTGTTTTTATAGGTTCCC
SOCS5 3’UTR-F	GGCTCGAG AGATGCTGCCTGCTGTTAATC
SOCS5 3’UTR-R	ATGCGGCCGCGCAGGTTATCCCTGCTTCTTG
Primers for RT-qPCR	
RT-gga-miR-5 S	AACTGGTGTCGTGGAGTCGGC
gga-5s-rRNA-F	CCATACCACCCTGGAAACGC
gga-5s-rRNA-R	TACTAACCGAGCCCGACCCT
RT-gga-miR-365-3p	CTCAACTGGTGTCGTGGAGTCGGCAATTCAGTTGAGATAAGGAT
gga-miR-365-3p-F	GGTAGGTAATGCCCCTAAAAATCC
gga-miR-365-3p-R	ACTGGTGTCGTGGAGTCGGC
GAPDH-F	CCTCTCTGGCAAAGTCCAAG
GAPDH-R	TTGATGTTGCTGGGGTCACG
SOCS5-F	GGCACTACCAGTACAAAAGCATC
SOCS5-R	CAAGGACCTGCGACTCGAAC
STAT1-F	CTTGATGCTGGGAGAGGAGT
STAT1-R	TGAGGGAGAGAGAGCGAAAG
STAT2-F	AGCAAAACTGTCCTGGTTGG
STAT2-R	GACCCTCATTGGTGCCTCT
STAT3-F	AAGCGTGGTCTCAGCATTGA
STAT3-R	CCAGCCAGACCCAGAAAGAG
STAT4-F	TGAAAGCAATCTGGGTGGA
STAT4-R	TCGCAGTATGTCAGCAAAGG
STAT5-F	TTGACCTGGACGACACCAT
STAT5-R	GACACAAACACGGCACAGTC
STAT6-F	CCTGAAGAGCTACTGGTCGG
BCL2-F	AAGCAAGCGTGACAAC
BCL2-R	ATCATAGGCTGCACATAC
BCLXL-F	CGGCTCATCATCCAGTCC

**Table 2:** **Sequences of RNA oligonucleotides**

Name	Sequences (5′–3′)
miR-365-3p mimics	UGUGUGCAACUACAGAUUGC
	AAUGCAACUACAAUGCACUU
miR-365-3p NC	UUCUCCGAACGUGUCACGUTT
	ACGUGACACGUUCGGAGAATT
miR-365-3p inhibitor	GCAAUUGCCUACAAUGCACAU
miR-365-3p inhibitor-NC	CAGUACUUUUGUGUAGUACAA

### Construction of 3’-UTR-luciferase plasmid and dual-luciferase reporter assay

The wild-type and mutant 3’-UTR DNA fragments of SOCS5 covering the predicted binding sites of gga-miR-365-3p were successfully cloned. The psiCHECK™-2-SOCOS-3’UTR (wild type and mutant) vector was constructed by combining the luciferase vector psi-CHECK™-2 (Promega, Madison, WI, USA) with SOCOS 3’-UTR (wild type and mutant).

Dual-luciferase reporter assay, as we have reported before [19]. In a nutshell, when cells reached 80–90% confluence, using Lipofectamine 2000 (Invitrogen Life Technologies, USA) each co-transfected cells with wild-type or mutant reporter plasmid (200 ng) and 10 pmol of the indicated RNA oligonucleotides. Then, the luciferase activity in each group was detected using an automatic microplate reader (Bio-Rad, Hercules, CA, USA) in accordance with the dual luciferase reporter gene detection kit instructions (Promega, Madison, WI, USA) according to the manufacturer’s protocol.

### Overexpression or inhibition of gga-miR-365-3p

Once 80–90% confluence was achieved, each group of cells was transfected with gga-miR-365-3p -mimics, gga-miR-365-3p-mimics-NC, gga-miR-365-3p-inhibitor, and gga-miR-365-3p-inhibitor-NC, respectively. The CP-II cells transfected with gga-miR-365-3p-mimics were marked as miR-365; cells transfected with gga-miR-365-3p-inhibitor were marked as miR-365-Inh; cells transfected with a non-specific RNA were marked as miR-NC or miR-Inh-NC. After 48 h transfection, TRNzol (TIANGEN, Beijing, China) was used to extract RNA from cells and subsequently detect the level of gga-miR-365-3p using qPCR.

### RNA isolation and quantitative real-time PCR

According to the manufacturer’s instructions, total mRNA was isolated from post-infected and non-infected cells via TRNzol Universal Reagent kit (TIANGEN, Beijing, China). Then, RNA was inverse transcribed to cDNA with the first strand cDNA synthesis kit (Cat No.11,119–11,141; Yeasen, Shanghai, China) and performed reverse transcription PCR (RT-PCR).

### Cell proliferation and apoptosis assays

The Cell Counting Kit-8 (CCK-8, DOJINDO, Shanghai, China) was used for cell proliferation experiments. CP-II cells were inoculated on a 96-well plate at 2 × 10^4^ cells per well. Each group of cells were separately transfected with different oligonucleotides (gga-miR-365-3p, gga-miR-365-3p-NC, gga-miR-365-3p-inhibitor, gga-miR-365-3p-inhibitor-NC, Si-SOCS5, Si- SOCS5-NC) or plasmids (pcDNA3.1-empty, pcDNA3.1- SOCS5) using Lipofectamine^TM^3000 (Invitrogen Life Technologies, USA) and each group had 6 biological replicates. Next, MG-HS (7 µL, 10^10^ CCU/mL) was utilized to infect CP-II cells for 2 h. At 12 h, 24 h, and 36 h post-transfection, a cell proliferation curve was measured by the CCK-8 kit according to the manufacturer’s instructions.

Transfection treatments were described above. Annexin V, FITC apoptosis detection kit (DOJINDO) was used to test the cell apoptosis. Each group was repeated three times.

#### ELISA

The grouping of transfection treatments is described above. Forty-eight hours after transfection, the supernatants were collected and the pro-inflammatory cytokine (IL-6 and TNF-α) levels were detected with enzyme-linked immunosorbent assay kits (Bio Legend, San Diego, CA, USA) according to the manufacturer’s directions.

### Western blot

The grouping of transfection treatments is described above. Forty-eight hours after transfection, the total proteins were extracted from CP-II cells, and their concentrations were then determined to use a Bicinchoninic acid (BCA) protein assay reagent kit (Transgen, Shanghai, China). Equal amounts of protein were separated by 12% SDS-polyacrylamide gel electrophoresis (Beyotime, China) and blocked with 5% skim milk for 1 h. Then, primary antibodies for SOCS5 (ABclonal, A7952), p-STAT1 (ABclonal, A19563), p-STAT3 (ABclonal, A19729), JAK (ABclonal, A11963), Bcl-2(ABclonal, A19693), Bcl-XL (ABclonal, A0209), Caspase3 (ABclonal, A19654) (all at 1:2000 dilution) and GAPDH (Abmart, M20024) or *β*-actin (Abmart, T40104) (at 1:5000 dilution) protein were incubated overnight at 4 ℃. Finally, the membrane was incubated with secondary antibody for 1 h after Tris Buffered Saline with Tween-20 (TBST) (A 1 × concentrated solution of Tris Buffered Saline with Tween-20 with a concentration of 10mM Tris. HCl, 15mM NaCl, 0.05% Tween-20 at pH7.5) washing. The enhanced chemiluminescence (ECL) detection system (Bio-Rad) was used to detect protein expression.

### Statistical analysis

Three independent duplicates were set in each experimental group, and the results were analyzed using GraphPad Prism 7. Multicomparison ANOVA, Tukey Method, was used as a statistic tool to compare the different experimental values; or the Student *t*-test for the comparison of two conditions. The data were expressed as the mean ± SD. *P* values < 0.05 were considered to have significant difference.

## Results

### Dysregulated gga-miR-365 expression after MG infection in CP-II cells

**Figure 1:**
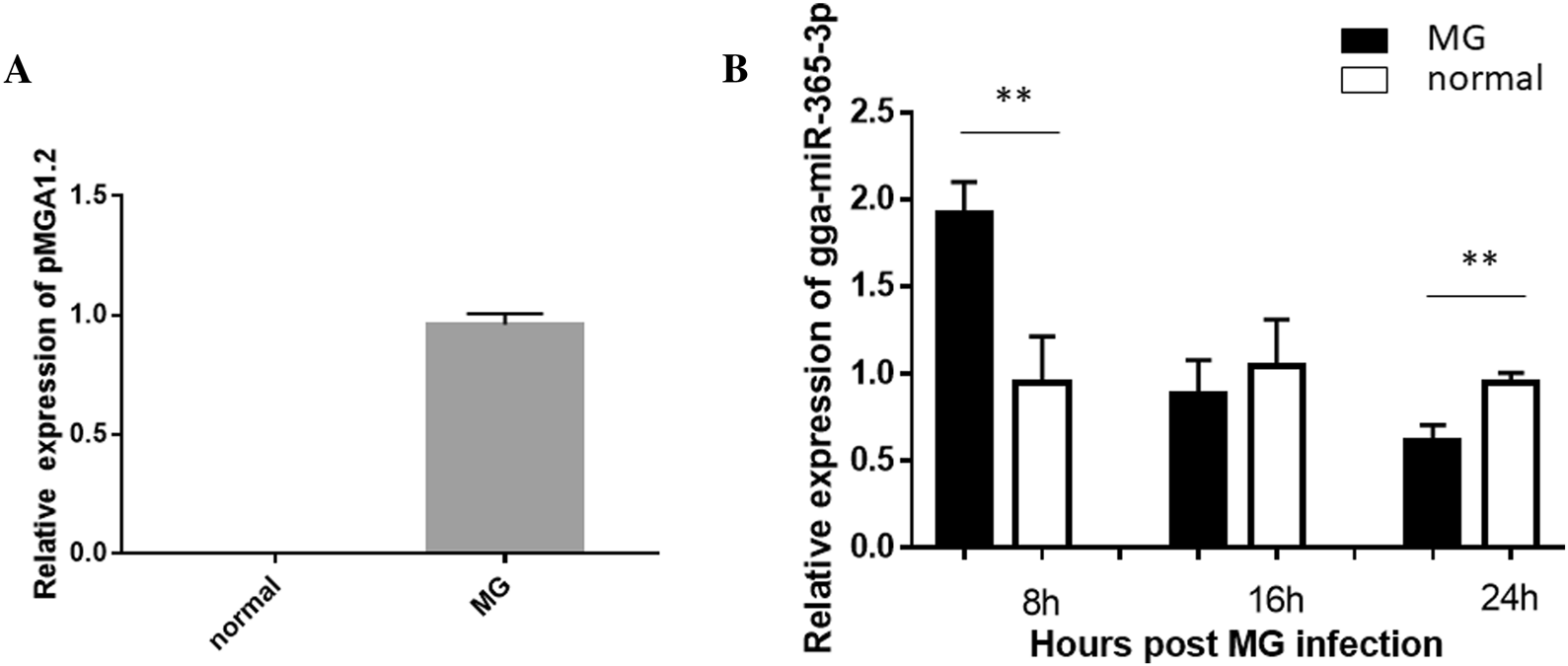
**Expression levels of gga-miR-365-3p in MG-infected CP-II cells. CP-II cells were separated and cultured in 6-well plates and treated MG. **After 24 h infection, the total RNA was isolated and the gga-miR-365-3p (**B**) or pMGA1.2 (**A**) expression was detected by qPCR. 5s-RNA or GAPDH was used as the internal quantitative control gene. All measurements shown are the means ± SD from three independent experiments, each with three replicates.

A model of MG-HS infection of CP-II cells was established according to the previous publication [34]. qPCR results show that MG infection of CP-II cells resulted in extremely high expression of pMGA1.2 mRNA, while the expression of pMGA1.2 mRNA could not be detected in normal cells (Figure 1A). Interestingly, gga-miR-365 was significantly upregulated when CP-II cells were infected with MG for 8 h, whereas gga-miR-365 was significantly downregulated when CP-II cells were infected for 24 h using qPCR (Figure 1B).

### Gga-miR-365-3p down-regulates pMGA1.2 expression

**Figure 2:**
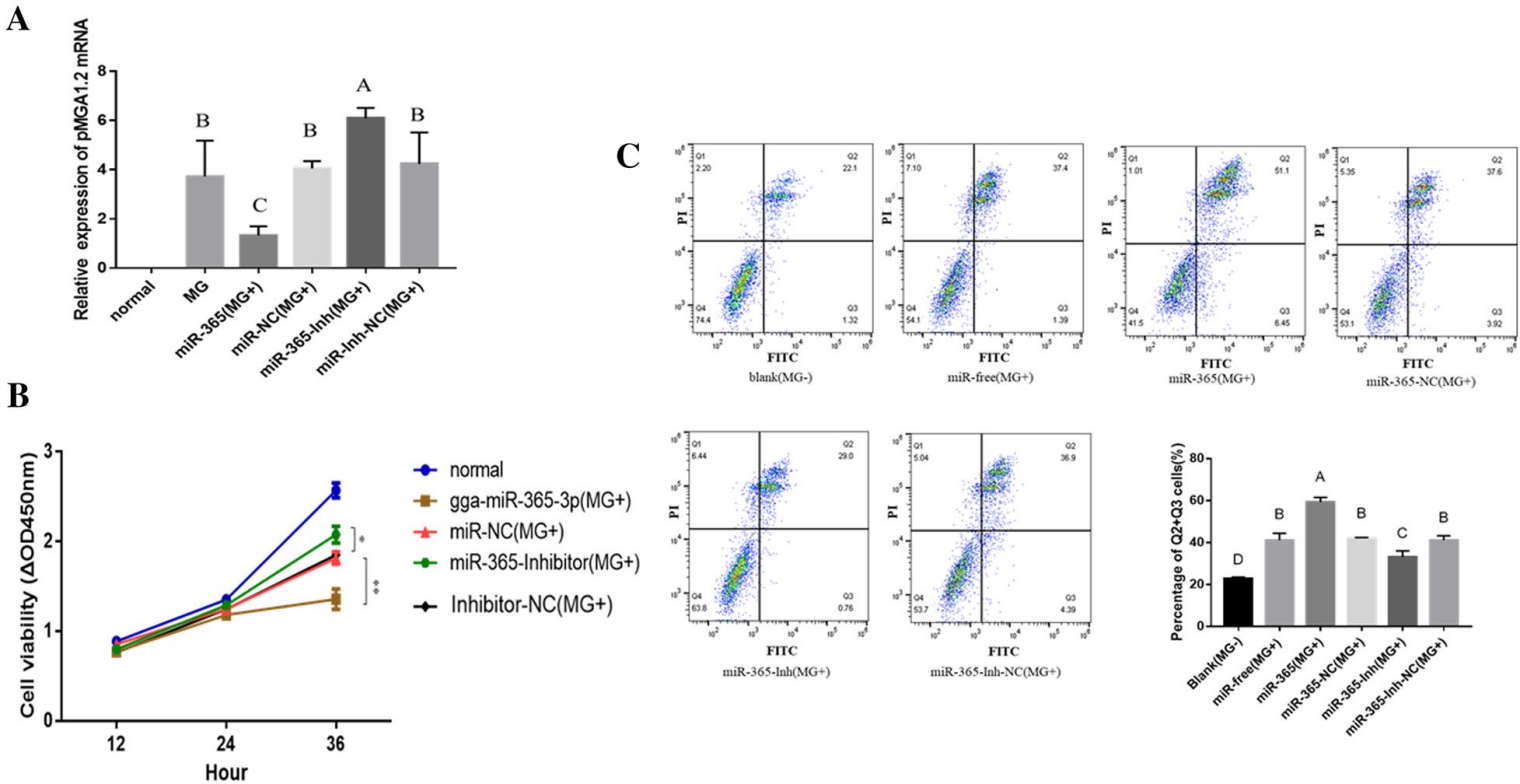
**
The effect of gga-miR-365-3p CP- cells. CP- cells were transfected with siRNA for 24 h and then infected with MG-HS (A).** qPCR was used to detect the mRNA expression of pMGA1.2. GAPDH was used as the internal quantitative control gene (**B**, **C**). CP cells were transfected with gga-miR-365-3p 50 nM mimics, 100 nM inhibitors, or their respective control and infected with 100 µL of MG-HS (1 × 10^10^ CCU/mL). Then CCK-8 kit was used to detect cell proliferation at different times (**B**). After 48 h of treatment, the CP- cells were stained with Annexin V– PI, and analyzed by flow cytometer. Apoptosis cell ratio is shown in (**C**).

To investigate the regulatory role of disturbed gga-miR-365-3p on MG infection, CP-II cells were transfected with oligonucleotides and then co-cultured with MG-HS for 24 h. We found that overexpression of gga-miR-365-3p was able to suppress the expression of pMGA1.2 mRNA, while the opposite result was obtained when gga-miR-365-3p was suppressed (Figure 2A). These results indicate that gga-miR-365-3p could inhibit the adhesion of MG to CP-II cells.

### Gga-miR-365-3p inhibits cell proliferation and promotes cell apoptosis in MG-infected CP-II cells

To further explore the potential functions of gga-miR-365-3p in MG infection, cell proliferation and apoptosis were examined both in over expression and loss-of-function studies for gga-miR-365-3p in CP-II cells. The cell growth assay shows that MG infection was extremely significant in inhibiting cell proliferation. Compared with the control group, gga-miR-365-3p mimics could result in decreasing the growth of MG-infection CP-II cells, whereas a significant increase in cell proliferation was observed when gga-miR-365-3p was inhibited (Figure 2B). As expected, MG infection with CP-II significantly increased the rate of apoptosis, and overexpression of gga-miR-365-3p could further increase apoptosis by flow cytometry analysis. In contrast, a gga-miR-365-3p inhibitor significantly inhibited the increase in apoptosis induced by MG infection (Figure 2C).

### Gga-miR-365-3p directly targets and negatively regulates SOCS5

**Figure 3:**
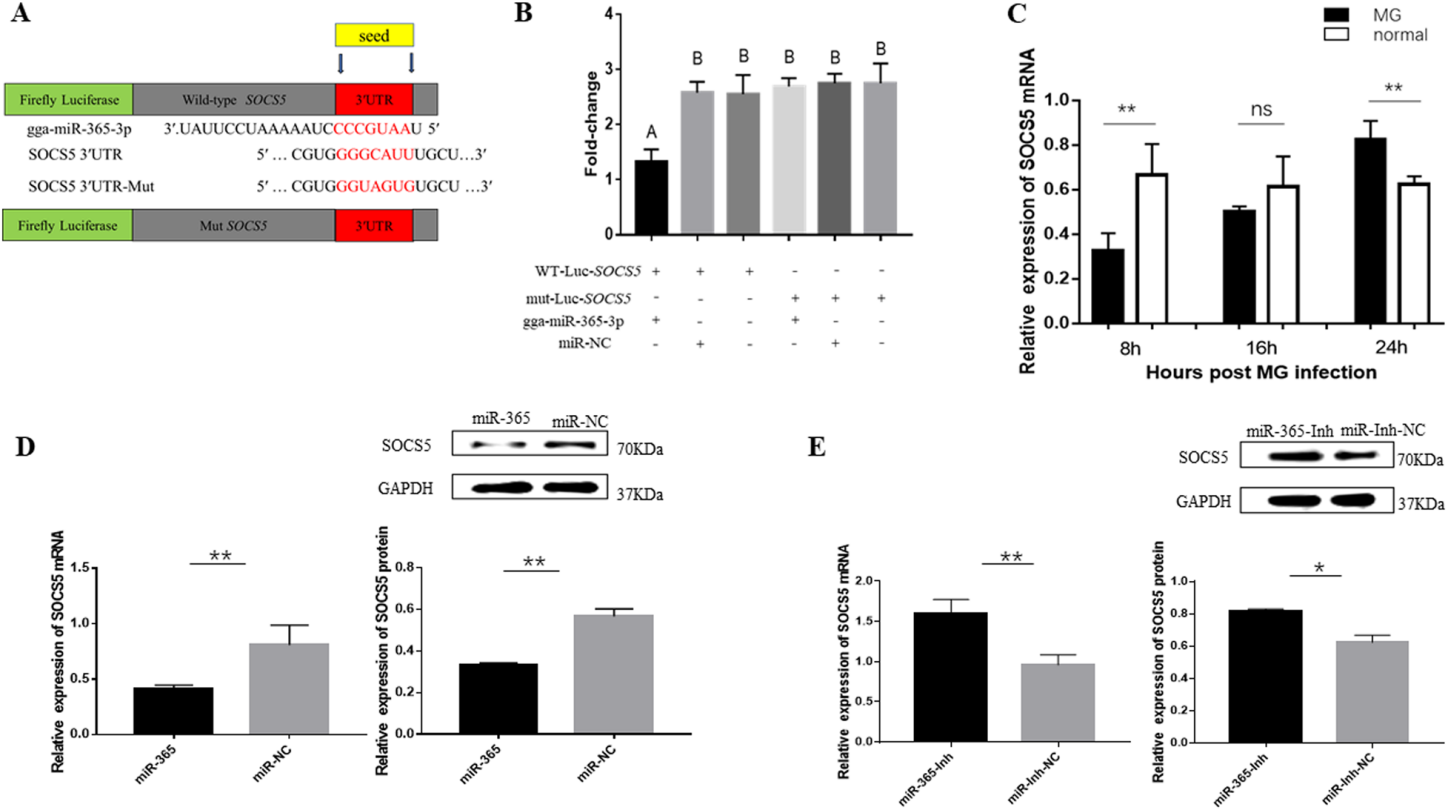
** gga-miR-365-3p directly targets SOCS5. A, B 24 h after co-transfection of dual-luciferase plasmid and siRNA, luciferase activity was measured using dual-luciferase assays.** **C** Expression levels of SOCS5 in MG-infected CP-II cells. **D**, **E** gga-miR-365-3p negatively regulates SOCS5. The expression of SOCS5 in different treatment groups was detected by qPCR or Western-blotting and normalized to GAPDH. Gray values of the indicated protein were measured by Fusion software. The data are the mean ± S.D. of at least 3 independent experiments.

It is well known that miRNA function by targeting and regulating target genes. Target gene prediction shows that SOCS5 might be a potential target gene of gga-miR-365-3p. To validate whether gga-miR-365-3p directly targets SOCS5, we conducted a luciferase reporter assay in CP-II cells. Compared with a control, gga-miR-365-3p mimics could significantly reduce the luciferase activity of the reporter containing wild-type 3’-UTR, while having no significant effect on the luciferase activity of mutant 3’ -UTR reporter (Figures 3A and B). It indicated that gga-miR-365-3p could be complementarily bound to 3’-UTR of SOCS5. Moreover, SOCS5 mRNA expression in CP-II cells after MG infection was at first sharply reduced and then upregulated, which was in contrast to the expression of gga-miR-365-3p (Figure 3C).

In addition, we also detected the expression of SOCS5 in CP-II cells treated with RNA oligonucleotides. As expected, gga-miR-365-3p mimics significantly inhibited both the expression of SOCS5 mRNA and SOCS5 protein, while gga-miR-365-3p inhibitor had the opposite effect (Figures 3D and E). Taken together, these results confirmed that SOCS5 was a direct target of gga-miR-365-3p and its expression was negatively regulated by gga-miR-365-3p.

### Gga-miR-365-3p activated the JAK/STAT signaling pathway by targeting SOCS5

**Figure 4:**
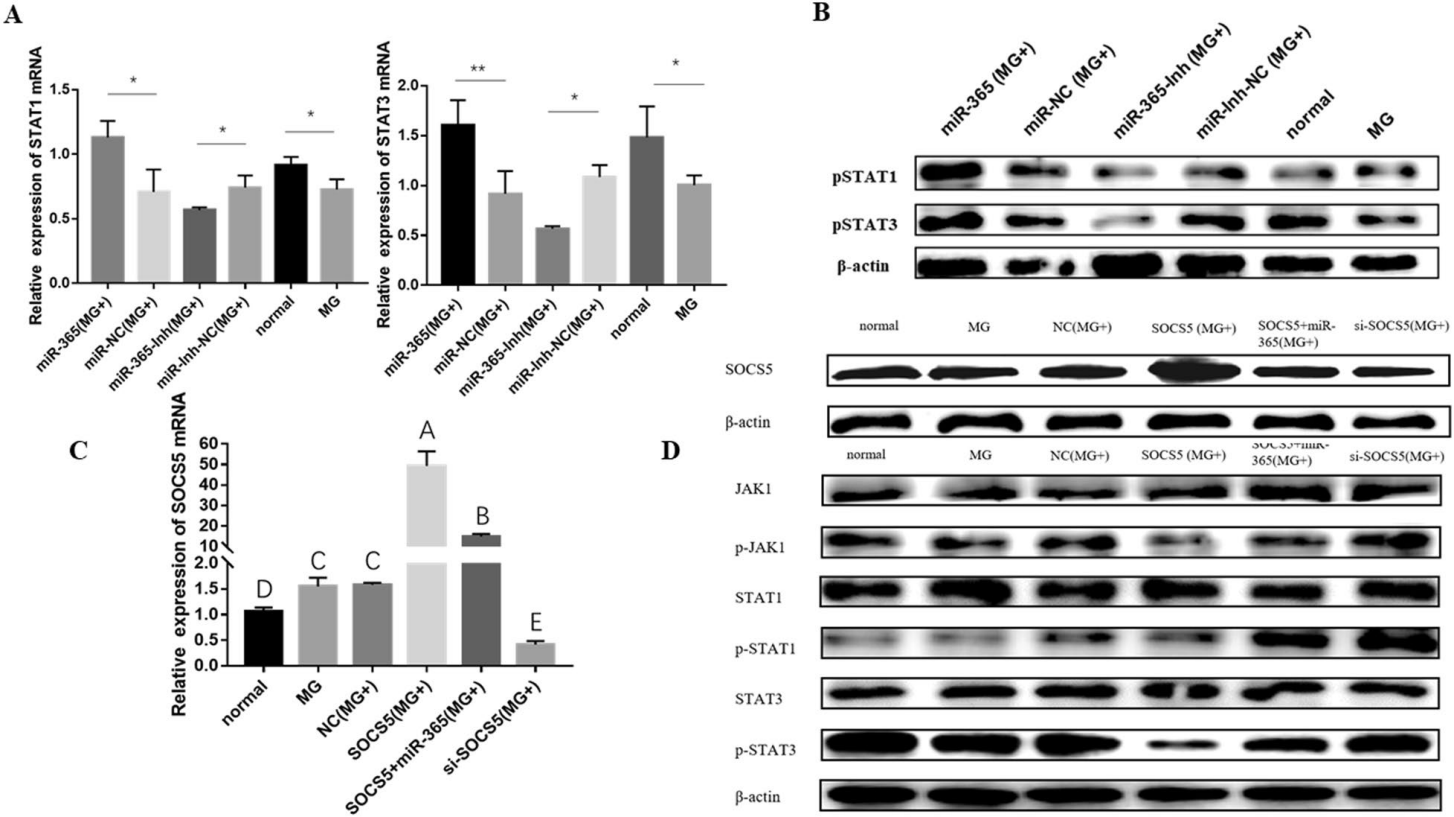
**The regulatory effect of gga-miR-365-3p on JAK/STAT pathway. After 12 h of transfection with synthesize RNA oligonucleotides, the CP-II cells were challenged by 100 µL MG-HS (1 × 10**^**10**^ CCU/mL). (**A**) The mRNA expression of STAT1 and STAT3. (**B**) The protein expression of STAT1 and STAT3. β-actin served as the loading control. (**C**) The mRNA expression of SOCS5 was detected by RT-qPCR. GAPDH works as a house-keeping gene. (**D**) The protein in SOCS5 and JAK/STAT signaling axis were detected by Western-blotting. *β*-actin was used for normalization. The data are the mean ± S.D. of at least 3 independent experiments.

SOCS, a STAT-mediated STAT repressor protein (SSI), is transcriptionally induced by an activated JAK/STAT pathway [35]. The above - described experiments indicate that SOCS5 is the direct target of gga-miR-365-3p. Therefore, to further understand the regulatory role of gga-miR-365-3p on SOCS5, we investigated the relationship between gga-miR-365-3p and JAK/STAT pathway. The qPCR and WB results show that the expression of STAT1 and STAT3 mRNA and phosphorylated protein were significantly decreased in the MG-infected group compared with the normal group. Overexpression of gga-miR-365-3p significantly increased the expression of STAT1 and STAT3 mRNA and phosphorylated proteins in MG-infected CP-II cells. Besides, there was an opposite result observed when gga-miR-365-3p was inhibited (Figures 4A and B).

Subsequently, overexpressed SOCS5 plasmid and SOCS5 small interfering RNA (Si-SOCS5) were successfully constructed and transfected into CP-II cells. The results show that SOCS5 overexpression plasmid was able to significantly increase SOCS5 levels, while si-SOCS5 was able to significantly decrease SOCS5 expression (Figures 4C and D). Then, upon overexpression or knockdown of SOCS5, the levels of genes related to the JAK/STAT pathway were detected by WB. WB show that the expression of p-JAK1, p-STAT1, and p-STAT3 proteins were significantly reduced in CP-II cells infected with MG after overexpression of SOCS5 compared to the control group [NC (MG+)]. To a certain extent, when gga-miR-365-3p and SOCS5 were co-overexpressed, the inhibitory effect of SOCS5 on p-JAK1, p-STAT1 and p-STAT3 was rescued. We also found that p-JAK1, p-STAT1 and p-STAT3 protein expression were significantly enhanced in the gga-miR-365-3p overexpression group [miR-365 (MG+)] or the SOCS5 knockdown group [si-SOCS5 (MG+)] (Figure 4D). Together, these results demonstrate that gga-miR-365-3p activated the JAK/STAT signaling pathway by targeting SOCS5.

### Gga-miR-365-3p down-regulates BCLXL and BCL2 expression levels and promotes caspase3 activation by targeting SOCS5

**Figure 5:**
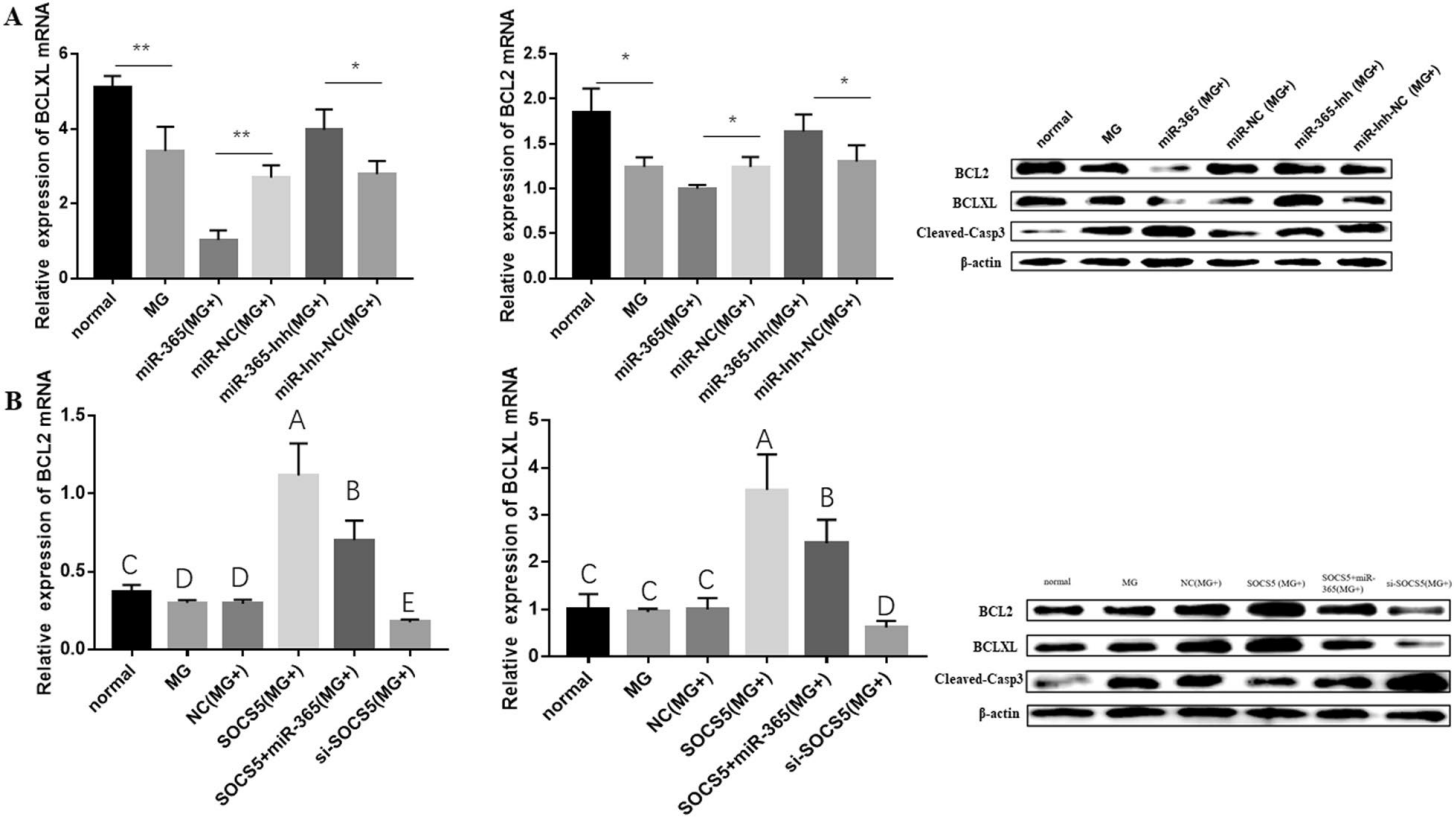
**The regulatory effect of gga-miR-365-3p/SOCS5 on apoptosis related genes.** After 12 h of transfection with synthesized RNA oligonucleotides or plasmids, the CP-II cells were challenged by 100 µL MG-HS (1 × 10^10^ CCU/mL). RT-qPCR was used to detect the relative expression of BCLXL and BCL2 mRNA; WB was used to detect the protein levels of apoptosis related genes. β-actin was used for normalization. The data are the mean ± S.D. of at least 3 independent experiments.

Subsequently, we explored the role played by gga-miR-365-3p in regulating apoptotic genes. The qPCR and WB results show that both mRNA and protein expression of BCLXL and BCL2 were significantly decreased in the MG-infected group compared with the normal group, while the expression of cleaved-caspase3 protein was significantly increased. Overexpression of gga-miR-365-3p significantly suppressed the expression levels of BCL2 and BCLXL, but increased the protein levels of activated caspase3. As expected, gga-miR-365-3p inhibitor had an opposite effect (Figure 5A).

Meanwhile, our data revealed that overexpression of SOCS5 in CP-II cells treated with MG dramatically increased BCLXL and BCL2 mRNA and protein expression levels, while remarkably decreasing cleaved-caspase3 protein expression (Figure 5B). Besides, there was an opposite result observed when SOCS5 was knocked down. Moreover, the regulation of BCL2, BCLXL and caspase3 by SOCS5 was unsurprisingly rescued when gga-miR-365-3p was co-overexpressed with SOCS5. These results suggest that overexpression of SOCS5 could promote BCL2 and BCLXL expression and inhibit caspase-3 protein activation (Figure 5B). Overexpression of gga-miR-365-3p could selectively target SOCS5 to inhibit BCL2 and BCLXL expression and promote caspase-3 protein activation.

### Gga-miR-365-3p activates the JAK/STAT signaling pathway to reduce pMGA1.2 expression through targeting SOCS5

**Figure 6:**
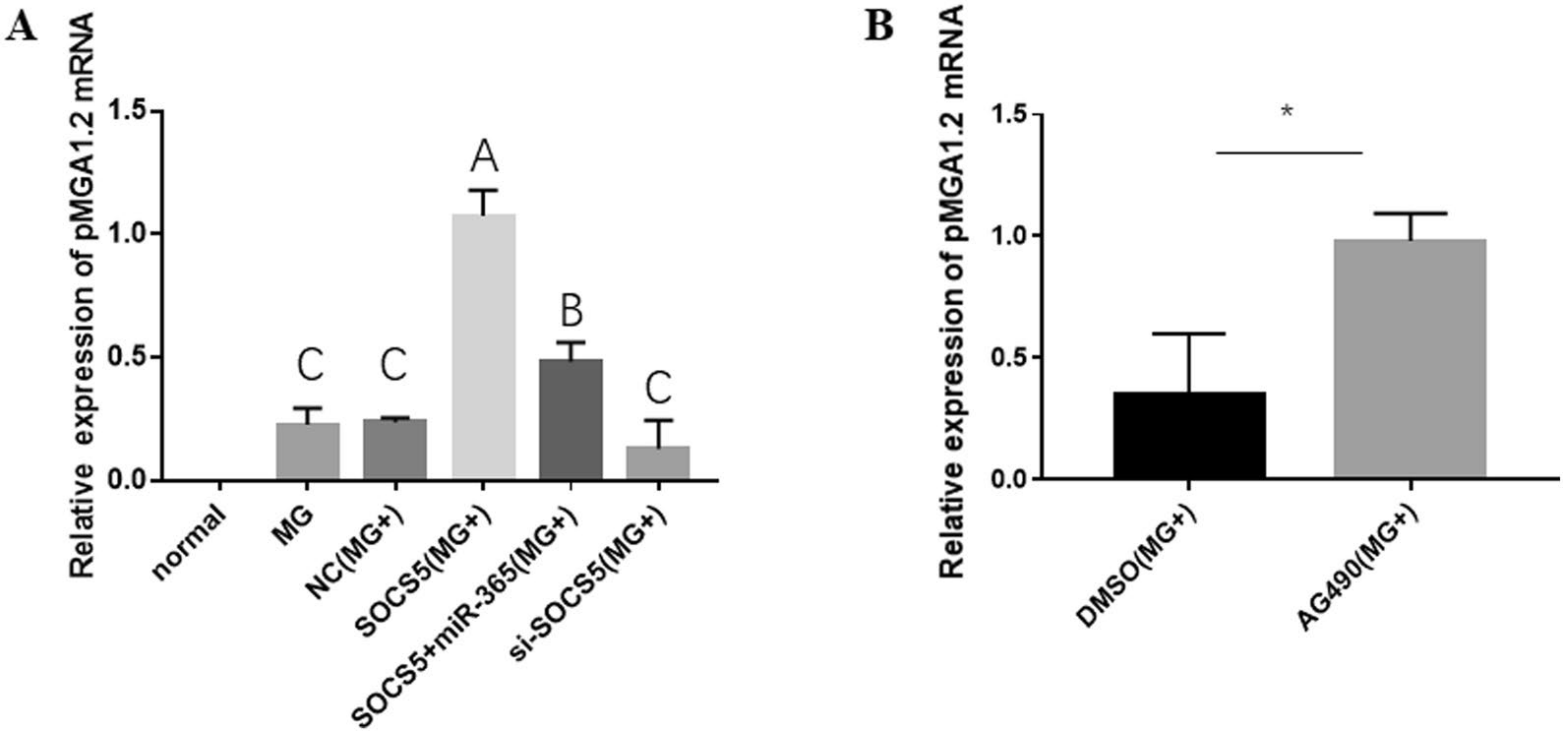
** Effect of SOCS5 and AG490 on pMGA1.2 levels in MG-infected CP-II cells (A, B).** The mRNA expression of pMGA1.2 was detected by RT-qPCR. GAPDH works as a house-keeping gene. The data are the mean ± S.D. of at least 3 independent experiments.

pcDNA3.1-SOCS5, SOCS5 siRNA, gga-miR-365-3p mimics or AG490 (a JAK/STAT pathway inhibitor) were transfected into CP-II cells infected with MG to further investigate the regulatory mechanism of gga-miR-365-3p on pMGA1.2. The qPCR results show that the expression of pMGA1.2 mRNA was significantly higher in the SOCS5 (MG+) and SOCS5 + miR-365 (MG+) groups than the NC (MG+) group (*P* < 0.01). pMGA1.2 was significantly lower in the SOCS5 and gga-miR-365-3p co-overexpression groups [SOCS5 + miR-365 (MG+)] than in the SOCS5 overexpression group [SOCS5 (MG+)]. In addition, its expression was reduced in the SOCS5 knocked down group [si-SOCS5 (MG+)] compared to the control group (Figure 6A). However, when the JAK/STAT pathway was inhibited, the expression of pMGA1.2 actually increased significantly (Figure 6B). These results illustrate that gga-miR-365-3p activated the JAK/STAT signaling pathway and reduced pMGA1.2 expression by targeting SOCS5 in MG-infected CP-II cells.

### Gga-miR-365-3p inhibits cell proliferation and promotes cell apoptosis through activating the JAK/STAT pathway by targeting SOCS5

**Figure 7:**
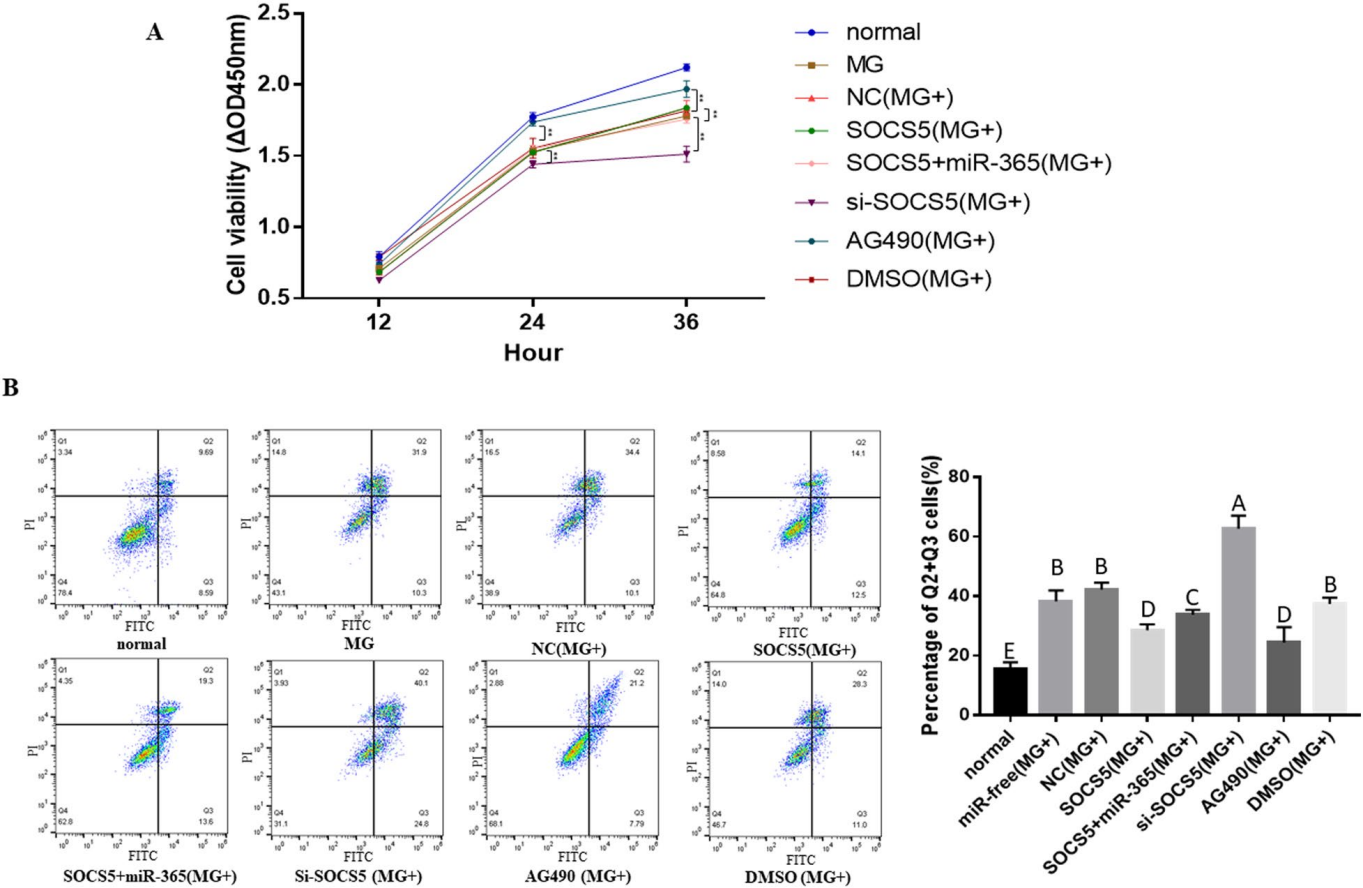
**Effects of SOCS5 and AG490 on the proliferation and apoptosis of CP-II cells infected with MG.** CP-II cells were transfected with gga-miR-365-3p mimics, over-SOCS5 plasmid, Si-SOCS5, or their respective control, and infected with MG-HS. (**A** Cell Counting Kit-8 was used to calculate cell proliferation results. **B** The cells were stained with Annexin V– PI, and analyzed by flow cytometer after 48 h post-infection. The data are the mean ± S.D. of at least 3 independent experiments.

To further demonstrate the effect of gga-miR-365-3p on the proliferation and apoptosis of MG-infected CP-II cells through the JAK/STAT pathway by targeting SOCS5, CP-II was transfected with pcDNA3.1-SOCS5, si-SOCS5, gga-miR-365-3p mimics or AG490, respectively. Then, we examined the effects of SOCS5 on CP-II cell proliferation and apoptosis. The assay shows that compared with the negative control group [NC (MG+)], cell proliferation was significantly reduced in the si-SOCS5 (MG+) group at both 24 and 36 h post-transfection. While overexpression of SOCS5 could remarkably increase the proliferation of MG-infected CP-II cells at 36 h post-transfection. Moreover, cell proliferation of MG-infected CP-II was highly significant at 24 and 36 h after AG490 treatment (Figure 7A).

Subsequently, cell apoptosis was analyzed by flow cytometry. Our results show that the apoptosis rate of the blank (MG-) group was significantly decreased compared with other challenge groups. The rate of apoptosis was highly significantly reduced in the SOCS5 (MG+) group compared to the NC (MG+) group and the SOCS5 + miR-365(MG+) group. Knockdown of SOCS5 significantly increased the apoptosis rate of MG-infected CP-II cells. More importantly, when the JAK/STAT pathway was inhibited, cell apoptosis was significantly reduced (Figure 7B). These results indicate that gga-miR-365-3p inhibits cell proliferation and promotes cell apoptosis by activating the JAK/STAT pathway by targeting SOCS5.

### Gga-miR-365-3p/SOCS5 regulates cellular inflammatory responses through the JAK/STAT pathway

**Figure 8:**
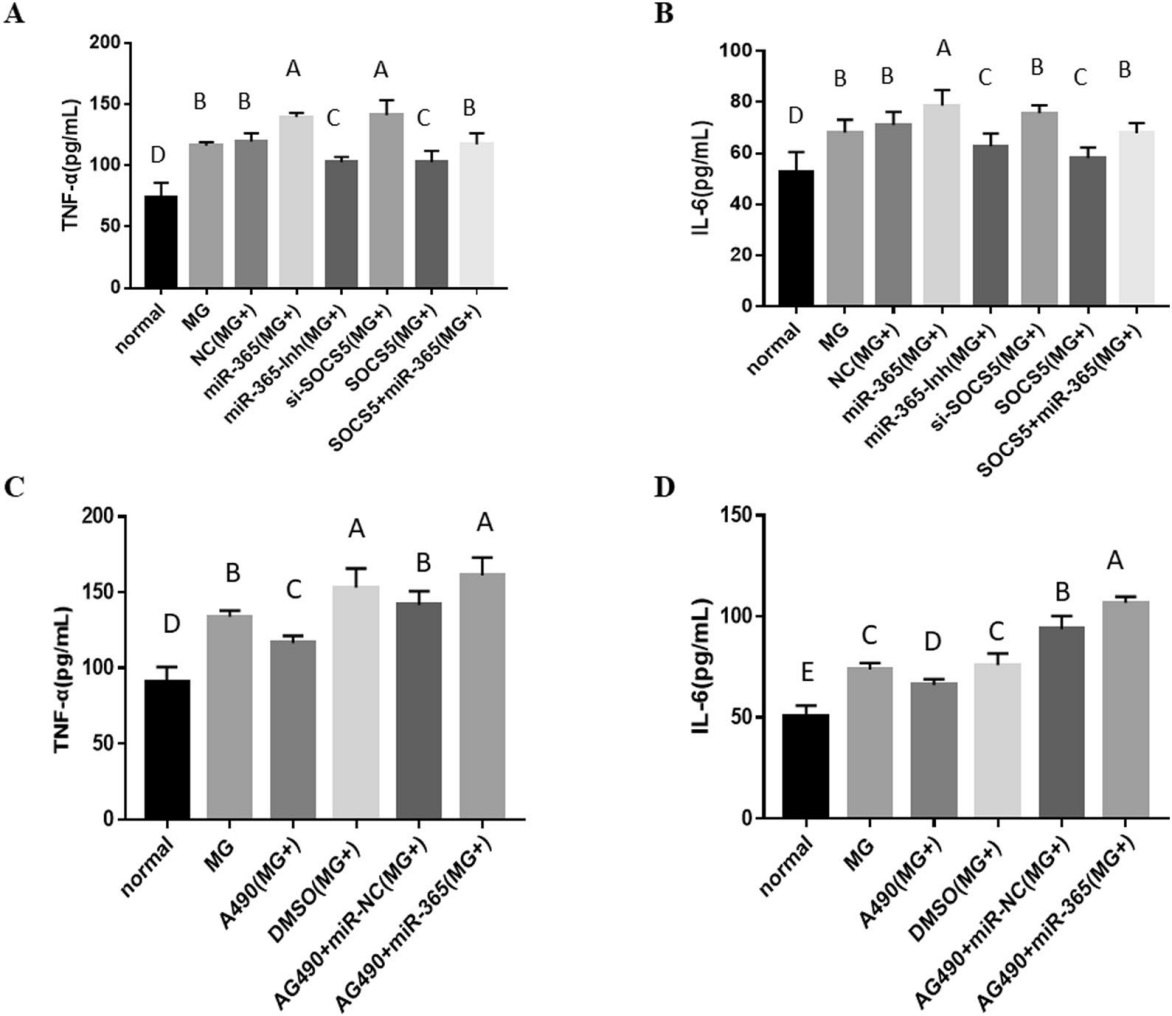
** The effect of gga-miR-365-3p, SOCS5 and AG490 on the secretion of TNF-α and IL-6 in CP-II cells infected by MG.** CP-II cells were transfected with gga-miR-365-3p mimics, over-SOCS5 plasmid, Si-SOCS5, or their respective control, and infected with MG-HS. The protein levels of pro-inflammatory cytokines (TNF-α and IL-6) were analyzed by ELISA. The data are the mean±S.D. of at least 3 independent experiments.

To investigate the effect of gga-miR-365-3p on the inflammatory response caused by MG infection, CP-II cells were transfected with pcDNA3.1-SOCS5, si-SOCS5, gga-miR-365-3p mimics or gga-miR-365-3p inhibitor, and cell supernatants were collected 24 h after treatment of cells. ELISA results show that TNF-α and IL-6 expression were highly significantly increased in the miR-365 (MG+) and si-SOCS5 (MG+) groups compared to the NC (MG+) group. Conversely, the TNF-α and IL-6 expression in the miR-365-Inh (MG+) and SOCS5 (MG+) groups were significantly lower than that in the NC (MG+) group. In addition, TNF-α and IL-6 release were significantly upregulated upon SOCS5 and gga-miR-365-3p co-overexpression compared to when SOCS5 was overexpressed (Figures 8A and B). Moreover, when the JAK/STAT pathway was inhibited, TNF-α and IL-6 were significantly reduced (Figures 8C and D). These results demonstrate that gga-miR-365-3p promotes cellular inflammatory responses through targeting SOCS5 to activate the JAK/STAT pathway.

### Inhibition of the JAK/STAT pathway reduces the expression of gga-miR-365-3p, overexpression of SOCS5 significantly increases the expression of gga-miR-365-3p in MG-infected CP-II

**Figure 9:**
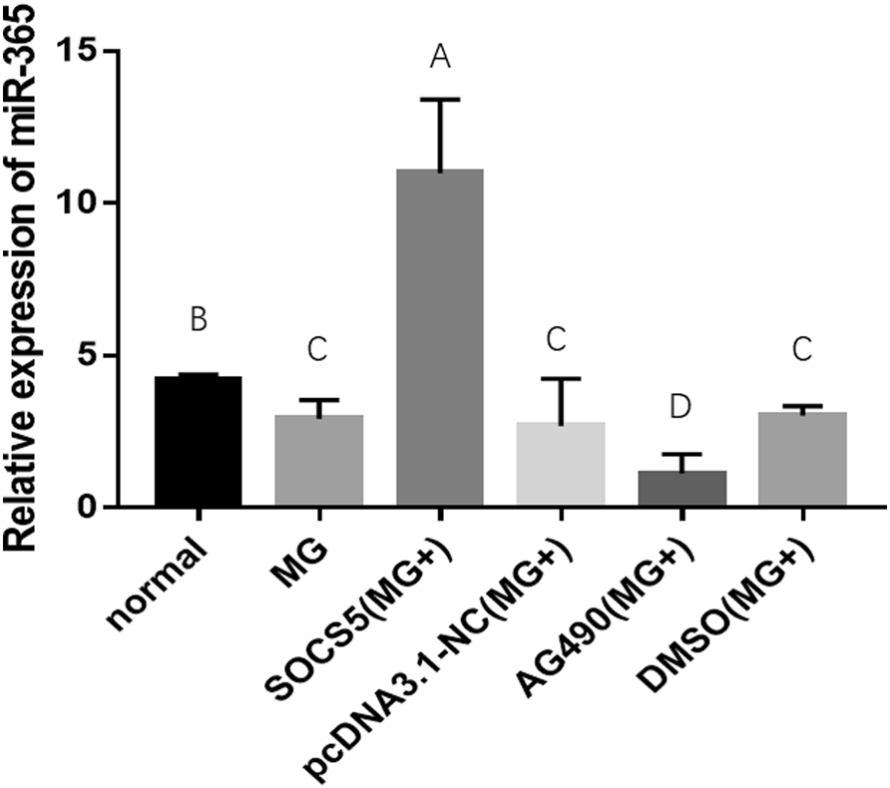
**
Relative mRNA expression gga-miR-365-3p with SOCS5 overexpression or AG490.** CP-II cells were transfected with over-SOCS5 plasmid, AG490, or their respective control, and infected with MG-HS. Then qPCR was used to detect the relative expression of gga-miR-365-3p. The data are the mean ± S.D. of at least 3 independent experiments.

To detect how SOCS5 and the JAK/STAT pathway influences the expression of gga-miR-365-3p, CP-II cells infected with MG were transfected with pcDNA3.1-SOCS5, AG490 or DMSO. After 24 h of cell treatment, compared with other groups, overexpression of SOCS5 could significantly increase the expression of gga-miR-365-3p. However, after treatment with AG490, compared with other groups, gga-miR-365-3p was significantly downregulated in MG-infected CP-II cells (Figure 9). This evidence suggests that gga-miR-365-3p expression was reduced by inhibiting the JAK/STAT signaling pathway to downregulate SOCS5 expression. There was negative feedback regulation between SOCS5 and gga-miR-365-3p.

## Discussion

Infection of MG induces a natural host immune response, and the JAK/STAT signaling pathway is essential in regulating the host immune response against pathogens such as bacteria, viruses, and parasites [36, 37]. Many reports have shown that miRNA regulate the JAK/STAT signaling pathway through inhibition of SOCS5 in response to pathogen infection. For instance, gga-miR-130b and gga-miR-454 target SOCS5 and SOCS6, respectively, to inhibit IBDV replication [16, 17]; miR-101 and miR-26a promote type I interferon to inhibit FHV-1 replication via SOCS5 [38, 39]. However, the role of SOCS5 in the process of MG infection remains unclear. In this study, gga-miR-365-3p increased rapidly in cells at the early stages of MG infection, which led to a decrease in SOCS5 expression. In contrast, MG down-regulated gga-miR-365-3p in cells at the late stage of infection, resulting in an increase in SOCS5 expression (Figures 1 and 3). SOCS is regulated by the JAK/STAT signaling pathway and constitutes a negative feedback loop through SOCS in JAK/STAT signaling that inhibits the immune response. In brief, activated STAT stimulate transcription of the SOCS genes; on the contrary, SOCS proteins bind phosphorylated JAK and their receptors to turn off the pathway [40, 41]. SOCS protein with negative feedback regulation prevents cytokine overproduction causing an inflammatory storm [42]. Also, suppression of the immune response diminishes host clearance of pathogens. For example, while SOCS1 expression prevents lethal inflammatory responses induced by Chlamydia pneumoniae STAT1 and IFN-α/β, inhibition of IFN-α/β impedes host bacterial clearance [43]. In the present study, MG infection caused the secretion of pro-inflammatory factors that produced inflammation. Inhibition of gga-miR-365-3p and overexpression of SOCS5 reduced cellular secretion of pro-inflammatory factors TNF-α and IL-6 and boosted intracellular pMGA1.2 expression, which resulted in reducing cellular inflammatory damage while also facilitating MG survival (Figures 2 and 8).

pMGA1.2 plays a crucial role in the process of adhesion and invasion of host cells by MG-HS, and thus is significant for its pathogenic mechanisms in host cells [5]. In this study, we found that gga-miR-365-3p was significantly upregulated in infected cells compared with normal cells at the early stage of infection (8 h), but significantly decreased at the late stage of infection (24 h), whereas the expression level of SOCS5 was the opposite (Figures 1 and 3). Gga-miR-365-3p was able to significantly reduce intracellular pMGA1.2 expression, while SOCS5 was able to increase pMGA1.2 expression, which may be a means for MG to promote its own adhesion or invasion of cells (Figures 2 and 6). Studies have demonstrated that MG can invade erythrocytes and eukaryotic cells, with a low rate of invasion before 8 h and a significant increase in invasion by 24 h [44]; The mRNA levels of IFN-γ, IL-6, IL-1β and CCL20 peaked at 6 h and then decreased after MG infection in tracheal epithelial cells and HD-11 cells, while IFN-γ and IL-6 reached baseline levels at 24 h [45]. In this manuscript we obtained similar results, that is, the level of SOCS5 expression in CP-II cells was significantly reduced at 8 h of MG infection, leading to a large secretion of the inflammatory factors TNF-α and IL6, while at 24 h after MG infection in CP-II cells, SOCS5 expression was significantly increased, which in turn inhibited the secretion of pro-inflammatory factors (Figure 8). This may indicate that in the early stages of infection, a small amount of MG adhesion or invasion triggers the inflammatory response of cells, while in the later stages of infection development, MG invades cells in large numbers to disrupt their immune response and aggravate the infection.

It has been shown that transcription factors and miRNA regulate each other to form a feedback loop, which can be divided into positive and negative feedback loops according to the mode of action [46, 47]. Yamakuchi et al. reported that p53 can increase the p53 activity by inducing miR-34a expression and inhibiting SIRT1, which in turn increases the p53 activity to form a positive feedback loop [48]. Aguda et al. found that a negative feedback loop consisting of miR-145, Myc and p53 plays an important role in coordinating cell differentiation and proliferation processes in neural stem cells [49]. Interestingly, in MG-infected CP-II cells, we found that overexpression of SOCS5 resulted in a highly significant increase in gga-miR-365-3p expression, whereas blockade of JAK/STAT pathway signaling by AG490 resulted in a decrease in SOCS5 and gga-miR-365-3p expression (Figure 9). It may predict that SOCS5 overexpression may affect gga-miR-365-3p expression by regulating a certain transcription factor to form a feedback loop to maintain cellular immune homeostasis. Among infected CP-II cells, MG downregulated gga-miR-365-3p to increase SOCS5 expression, while enhanced SOCS5 in turn upregulates gga-miR-365-3p to form negative feedback, which may reveal a game between the host and the pathogen.

In summary, at the initial stage of MG invasion into cells, up-regulated gga-miR-365-3p activated the JAK/STAT signaling pathway through inhibition of SOCS5, inhibited proliferation of infected cells, promoted apoptosis, and contributed to increased secretion of inflammatory factors, activating the immune response against MG infection. At the late stage of MG invasion into cells, MG promoted proliferation of infected cells, inhibited apoptosis, and reduced secretion of inflammatory factors by reducing intracellular gga-miR-365-3p expression and inhibiting the JAK/STAT pathway to promote adhesion or invasion of cells to evade the host immunity. This work confirms the role of gga-miR-365-3p in MG infection and further reveals a feedback loop between gga-miR-365-3p and JAK/STAT signaling pathway involving molecular mechanism for fine regulating inflammation and immune system disorders of MG infection.
